# Prior knowledge driven Granger causality analysis on gene regulatory network discovery

**DOI:** 10.1186/s12859-015-0710-1

**Published:** 2015-08-28

**Authors:** Shun Yao, Shinjae Yoo, Dantong Yu

**Affiliations:** 1 Department of Biochemistry and Cell Biology, Stony Brook University, Stony Brook, 11790 NY USA; 2 Computational Science Center, Brookhaven National Laboratory, Upton, 11793 NY USA

**Keywords:** Time series, Gene expression data, Granger causality, Gene regulatory networks

## Abstract

**Background:**

Our study focuses on discovering gene regulatory networks from time series gene expression data using the Granger causality (GC) model. However, the number of available time points (*T*) usually is much smaller than the number of target genes (*n*) in biological datasets. The widely applied pairwise GC model (PGC) and other regularization strategies can lead to a significant number of false identifications when *n*>>*T*.

**Results:**

In this study, we proposed a new method, viz., *CGC-2SPR* (CGC using two-step prior Ridge regularization) to resolve the problem by incorporating prior biological knowledge about a target gene data set. In our simulation experiments, the propose new methodology *CGC-2SPR* showed significant performance improvement in terms of accuracy over other widely used GC modeling (PGC, Ridge and Lasso) and MI-based (MRNET and ARACNE) methods. In addition, we applied *CGC-2SPR* to a real biological dataset, i.e., the yeast metabolic cycle, and discovered more true positive edges with *CGC-2SPR* than with the other existing methods.

**Conclusions:**

In our research, we noticed a “ 1+1>2” effect when we combined prior knowledge and gene expression data to discover regulatory networks. Based on causality networks, we made a functional prediction that the *Abm1* gene (its functions previously were unknown) might be related to the yeast’s responses to different levels of glucose. Our research improves causality modeling by combining heterogeneous knowledge, which is well aligned with the future direction in system biology. Furthermore, we proposed a method of Monte Carlo significance estimation (*MCSE*) to calculate the edge significances which provide statistical meanings to the discovered causality networks. All of our data and source codes will be available under the link https://bitbucket.org/dtyu/granger-causality/wiki/Home.

## Background

Technology advances in molecular biology, especially those in Next Generation Sequencing (NGS) have innovated the principles of biology research [1]. These novel approaches have enabled biologists to perform high throughput parallel experiments efficiently in terms of time and cost. However, to analyze such data became a new challenge in the field of bioinformatics [2]. In all data mining tasks, gene regulatory network (GRN) inference and prediction has become one of the most exciting topics in the current era [3]. After sequencing and identifying the genes of an organism, researchers then became interested in how the genes regulate each other. The results of the recently finished ENCODE project further highlighted the importance of GRNs by revealing that most non-coding DNAs are involved in regulating gene expressions [4]. In short, research on GRN inference will continue to be important in the coming decade.

Although there are numerous studies on GRN inference, only a few of them have focused on the newly emerging type of data: i.e. time series gene expression data. With the help of NGS technology, researchers, for example [1], can easily acquire this type of data from a biological process, e.g., the cell cycle. Since nearly every individual biological phenomenon is a dynamic process involving in a time domain, biologists have become increasingly interested in collecting and analyzing time series gene expression data. Indeed, the well-known Gene Expression Omnibus (GEO) database has collected more and more time series data in recent years [5]. Thereby, developing reliable GRN inference strategies and algorithms for time series gene expression data has become an emergent task.

One popular approaches for predicting gene regulatory networks from time series data is Dynamic Bayesian Network (DBN) modeling. Early DBN methods rested on Boolean Network (BN) theories. Existing BN inference approaches include the REVEAL algorithm [6], the MDL algorithm [7] and other methods incorporating prior knowledge [8, 9]. Later studies on DBN models have also employed Gaussian distribution and BIC to model the continuous expression values, which yielded better results, though with a higher computation cost [10–13]. The DBN method performs well in general, but it can only handle a limited number of genes (network size) due to the combinatorial complexity in the model’s searching space [13, 14].

Furthermore, mutual information (MI) methods based on information theory were applied in a few studies [15, 16]. The networks resulting from these mutual information methods are usually non-directional. However, several recent studies have also utilized time delay MI methods to successfully generate small-scale directional networks [17–19].

Due to the increased expression data dimension size in recent years, another family of methods came into focus: Vector AutoRegressive (VAR) methods. Granger causality inference is one of the most popular VAR methods, originally proposed in economic studies [20, 21], and now introduced to gene regulatory network inference. Recently, researchers have compared the Granger causality approach with DBN methods using various models [13]. They discovered that Granger causality inference has a similar performance to that of DBN methods, but is much faster than DBN. With a growing number of genes to analyze, Granger causality inference will be the more preferable method to use due to its computational efficiency.

Nevertheless, there are still problems when traditional Granger causality inference is applied to biological gene expression data that have a common property: Data often contain a large number of genes (*n*) but an extremely small number of time points (*T*). On the one hand, pairwise Granger causality (PGC) model [22, 23] has been tried in considering two genes at a time. The limitation of data along the time dimension makes the PGC model susceptible to random noises. In addition, previous studies showed that PGC model could not differentiate direct causalities from indirect causalities effectively [24]. On the other hand, conditional Granger causality (CGC) model was also utilized and improved in different studies [22–25]. These improvements included the incorporation of different regularization terms to control the prior distribution of the regression parameters. However, these regularization methods based on non-informative priors did not add new information to the gene expression data, and thereby resulted only in a limited improvement.

It is noteworthy that there are still many types of traditional biological experiment data available in addition to those from high throughput experiments. To improve the performance, it is natural to incorporate such prior knowledge into GC modeling. Studies in Boolean Network (BN) inference have utilized prior knowledge to constrain the connectivity of nodes, so reducing the searching space [9]. Further, prior knowledge could also be used in DBN modeling to define the prior likelihood of a model [26]. However, these earlier methods cannot efficiently handle unbalanced time series data with lots of genes and few time points. To the best of our knowledge, no previous researches have tried to use prior knowledge to guide GC analysis.

In this paper, we offer the following contributions to handle the problem of unbalanced datasets (*n*>>*T*): First, we investigated the effects from unbalanced datasets based on simulations, and showed that none of the previous works performs well under *n* > > *T*. At the *n* > > *T* condition, the performance of pairwise models, including the PGC and MI methods deteriorates significantly. Moreover, simply incorporating regularization terms will not lead to a noticeable improvement. Our research is the first attempt to investigate the *n*>>*T* condition thoroughly in the field. Second, to overcome this problem, we propose to combine other evidence or prior knowledge into the conditional Granger causality analysis, termed **CGC-2SPR**, i.e. CGC using a two-step prior Ridge regularization. When prior knowledge is naively incorporated into regularization, positive and negative regulations cannot be distinguished properly. Our two-step procedure guarantees the correctness of integrating the prior knowledge in both positive and negative regulatory relationships. Third, we cannot simply use *F*-tests or other statistical tests due to *n*>>*T*. Then, we propose a Monte Carlo method, namely **MCSE**, to estimate significance levels, and obtain reasonable results when *n*>>*T*. Finally, we applied *CGC-2SPR* to a real biological dataset (yeast metabolic cycle gene expression data) and discovered a significantly larger number of known gene regulations than those revealed by the baseline methods, including PGC, MI modeling, and simple regularization methods.

## Methods

In this section, we propose a new methodology of applying Granger causality modeling to time series gene expression data. Firstly, we introduce two general GC modeling strategies as the foundation of our methods. Then, we illustrate the problems of applying general strategies and regularization methods to real gene expression data where *n*>>*T*. Lastly, we propose our new method *CGC-2SPR* and implement an efficient algorithm to apply the new method.

### Granger causality modeling

Granger causality proposed in the 1960s [20, 21], has proven to be an operational notion of causality (w.r.t. true causality) in time series data analysis. If there is a causal relationship between two random variables, the past values of the “cause” variable will contribute to predicting the future values of the “effect” variable.

#### Bivariate Granger causality modeling

To describe Granger causality modeling mathematically, we assume there are two time series data *y*_1,*t*_ and *y*_2,*t*_ with the same time length *T*. For simplicity, we simultaneously consider two Granger causalities *y*_1_⇒*y*_2_ and *y*_2_⇒*y*_1_. The maximum *lag* allowed in the past observation values is defined as a model order (*p*). The regressions for two random variables can be written as the following two equations (*t*=*p*+1,*p*+2,...,*T*):

(1) $$  \begin{aligned} y_{1,t}=\sum_{i=1}^{p}(a_{1,1,i}y_{1,t-i}+a_{2,1,i}y_{2,t-i})+e_{1,t} \\ y_{2,t}=\sum_{i=1}^{p}(a_{1,2,i}y_{1,t-i}+a_{2,2,i}y_{2,t-i})+e_{2,t} \end{aligned}  $$

where *e*_1,*t*_ and *e*_2,*t*_ represent the residuals of the two random variables, and *a* represents the model’s regression coefficients that can be re-written together in a 2×2 matrix form:

$$A_{i}=\left[ \begin{array}{cc} a_{1,1,i} & a_{2,1,i} \\ a_{1,2,i} & a_{2,2,i} \end{array} \right], i=1,2,...,p. $$

Together, *m*=*T*−*p* pairs of equations exist in the model (1), and can be represented together as a single matrix form:

(2) $$  Y=XB+E  $$

where

$$\underset{(m\times2)}{Y}=\left[ \begin{array}{cc} y_{1,p+1} & y_{2,p+1} \\ y_{1,p+2} & y_{2,p+2} \\ \vdots & \vdots \\ y_{1,T} & y_{2,T} \end{array} \right], $$

$$\underset{(m\times2p)}{X}=\left[ \begin{array}{ccccccc} y_{1,p} & y_{2,p} & \cdots & y_{1,1} & y_{2,1} \\ y_{1,p+1} & y_{2,p+1} & \cdots & y_{1,2} & y_{2,2} \\ \vdots & \vdots & \ddots & \vdots & \vdots \\ y_{1,T-1} & y_{2,T-1} & \cdots & y_{1,T-p} & y_{2,T-p} \end{array} \right], $$

$$\underset{(2p\times2)}{B}=\left[ \begin{array}{c} A_{1}' \\ A_{2}' \\ \vdots \\ A_{p}' \end{array} \right], \underset{(m\times2)}{E}=\left[ \begin{array}{cc} e_{1,p+1} & e_{2,p+1} \\ e_{1,p+2} & e_{2,p+2} \\ \vdots & \vdots \\ e_{1,T} & e_{2,T} \end{array} \right]. $$

Also there are two regressions for time series that are based only on its own past value:

(3) $$  \begin{aligned} y_{1,t}=\sum_{i=1}^{p}a_{1,1,i}y_{1,t-i}+e_{1,t} \\ y_{2,t}=\sum_{i=1}^{p}a_{2,2,i}y_{2,t-i}+e_{2,t} \end{aligned}  $$

To compare the models (Eq. 1) and (Eq. 3), we can calculate the residual square sum (RSS) of these regressions. Assuming the residual *e*_1,*t*_ and *e*_2,*t*_ have a zero mean Gaussian distribution, the maximum likelihood estimation (MLE) of the regression coefficients could be obtained by the ordinary least square (OLS) calculation. In this case, the regression solution *B* (Eq. 2) is equivalent to optimizing the following function:

(4) $$  \hat{B}=\underset{B}{\operatorname{argmin}}\frac{1}{2}||Y-XB||_{F}^{2}  $$

or in matrix notations,

(5) $$  \hat{B}=\underset{B}{\operatorname{argmin}}\frac{1}{2}tr((Y-XB)^{T}(Y-XB)).  $$

When *X* ^*T*^ *X* is invertible, the estimation of *B* could be written as follows:

(6) $$  \hat{B}=(X^{T}X)^{-1}X^{T}Y.  $$

From the estimation of *B*, the *R* *S* *S*_1_ of the full model (Eq. 1) can be determined. Through a similar procedure, the *R* *S* *S*_2_ of the reduced model (Eq. 3) can be calculated as well. Based the *RSS* values from the models (Eq. 1) and (Eq. 3), the *F*-score can be calculated through the following equation:

(7) $$  F=\frac{(RSS_{2}-RSS_{1})/p}{RSS_{1}/(m-2p)}  $$

If the time series data come from two independent variables, the *F*-score will follow the *F*(*p*,*m*−2*p*) distribution. Thus statistical tests could be applied to determine whether or not the former model is significantly better than the latter one.

**Fig. 1:**
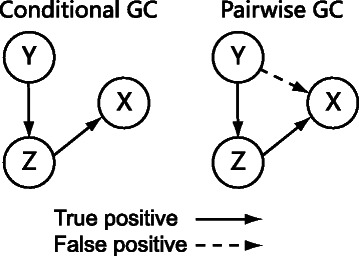
The application of PGC modeling will lead to high false discovery rate due to the existence of indirect causalities

In real biological research, when bivariate Granger causality model is used to deal with a target gene set with size *n*, all the possible causality pairs (*n*(*n*−1) directed pairs) are calculated independently. Thus, bivariate Granger causality model is also known as a pairwise Granger causality (PGC) model. Previous studies showed that PGC model suffers from a large number of false discoveries [24], due to the existence of indirect causalities (Fig. 1).

#### Multivariate Granger causality modeling

To mitigate the false discovery problem with the PGC model, we can extend a bivariate Granger causality modeling to a multivariate one in which three or more random variables of time series with *T* time points are considered (*n*≥3). Here, we use a single *n*×1 vector *y*_*t*_ to represent the observed values of all variables at time point *t*. In this case, the Granger causality model could be expressed as follows:

(8) $$  y_{t}=\sum_{i=1}^{p}A_{i}y_{t-i}+e_{t}, t=p+1,...,T.  $$

By stacking the equations together, we could obtain the same matrix as (Eq. 2):

(9) $$  Y=XB+E  $$

where

$$\underset{(m\times n)}{Y}=\left[ \begin{array}{c} y_{p+1}' \\ y_{p+2}' \\ \vdots \\ y_{T}' \end{array} \right], \underset{(m\times np)}{X}=\left[ \begin{array}{cccc} y_{p}' & y_{p-1}' & \cdots & y_{1}' \\ y_{p+1}' & y_{p}' & \cdots & y_{2}' \\ \vdots & \vdots & \ddots & \vdots \\ y_{T-1}' & y_{T-2}' & \cdots & y_{T-p}' \end{array} \right], $$

$$\underset{(np\times n)}{B}=\left[ \begin{array}{c} A_{1}'\\ A_{2}'\\ \vdots \\ A_{p}' \end{array} \right], \underset{(m\times n)}{E}=\left[ \begin{array}{c} e_{p+1}'\\ e_{p+2}' \\ \vdots \\ e_{T}' \end{array} \right]. $$

We could estimate the coefficient matrix *B* by OLS, the same as the bivariate GC model:

(10) $$  \hat{B}=(X^{T}X)^{-1}X^{T}Y.  $$

when *X* ^*T*^ *X* is invertible, in which case *m*≥*n* *p*.

Based on the estimation results, the Granger causality relationship between two variable *i* and *j* can be evaluated. With $\hat {B}$, we can calculate the *R* *S* *S*_*i*_ for *i*. We also could repeat the above process excluding variable *j* (totally (*n*−1) variables) and get another *R* *S* *S*_*ij*_ value. By constructing the *F* score from the two prediction errors, we also can statistically analyze the Granger causality relationship between variable *i* and *j* based on [24]. It is noteworthy that in the multivariate GC model, the causality relationship between the two variables is analyzed in the condition of the other (*n*−2) variables. Therefore, it is also referred as conditional Granger causality (CGC) model.

The key prerequisite to apply multivariate Granger causality model to a time series dataset is *m*≥*n* *p*. Considering *m*=*T*−*p*, the essential condition for applying multivariate Granger causality model is

(11) $$  T\geq (n+1)p.  $$

#### The problems of existing strategies

**Fig. 2:**
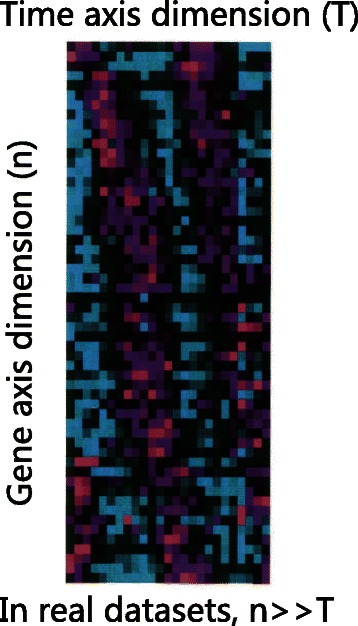
For real biological dataset [51], the number of genes (*n*) is far more than the number of time points (*T*), limiting the usage of CGC

Both Granger causality modeling methods have problems when they are applied to real biological datasets. Figures 1 and 2 show the problems of these two strategies. On one hand, earlier studies [24] revealed that the PGC model tends to generate more false discoveries than CGC model due to the existence of indirect causalities and random coincidences. The problem will deteriorate when the number of genes increases, and the true causality network becomes more complex. On the other hand, CGC model has its own limitations in that it requires enough data on the time dimension to satisfy the condition (Eq. 11), viz. that the number of time points exceeds the size of the target gene set. However, in real biological datasets, the number of random variables is much bigger than the number of time points *n*>>*T*. For instance, in the human HeLa cell cycle dataset *n*=1099 and *T*=47 [27] and in the yeast metabolic cycle dataset *n*=2935 and *T*=36 [28]. Therefore, it is a challenge to apply a solid traditional GC modeling approach to these real biological datasets. Next, we describe our new method *CGC-2SPR* to address the challenge.

### CGC-2SPR: Prior knowledge driven Granger causality modeling

To resolve the problems of applying the Granger causality model to real data with a limited number of time points, we proposed a new framework for the biological field to apply Granger causality analysis: Prior knowledge driven Granger causality modeling. In order to reduce the false discovery rate of PGC model, we consider all the genes together in the new framework as CGC model does. However, the standard CGC model cannot be applied here due to the aforementioned problem, and we must revise the CGC model to make it fit real biological data where *n*>>*T*.

As described in the previous section, CGC model could be viewed as an optimization problem (the same as Eq. 5):

$$\hat{B}=\underset{B}{\operatorname{argmin}}\frac{1}{2}||Y-XB||_{F}^{2} $$

where the matrix *Y*(*m*×*n*), *X*(*m*×*n* *p*) and *B*(*n* *p*×*n*) follow the same notation as in Eq. 9.

When *T*<(*n*+1)*p*, *Y*=*X* *B* is an under-determined problem and has an infinite number of solutions. However, with Ridge or Lasso regularization terms [29, 30] on the optimization function, the equation will have certain solutions with the desired properties. For instance, Ridge regularization gives preference to smooth solutions while Lasso regularization prefers sparse solutions. Using regularizations can mitigate the problem of overfitting.

Considering all the genes together with the Ridge or Lasso regularization, we can formulate two additional optimization problems:

(12) $$  {\small{\begin{aligned} Ridge\ regularization: &\\ \hat{B}=\underset{B}{\operatorname{argmin}}&\frac{1}{2}*||Y-XB||_{F}^{2}+\frac{1}{2}\lambda_{1}*||B||_{F}^{2} \\ Lasso\ regularization: &\\ \hat{B}=\underset{B}{\operatorname{argmin}}&\frac{1}{2}*||Y-XB||_{F}^{2}+\lambda_{1}||B||_{1}. \end{aligned}}}  $$

where ||*B*||_*F*_ is the “entrywise” *L*_2_-norm of matrix ||*B*|| (Frobenius norm), representing the square root of the element square summation and ||*B*||_1_ is the “entrywise” *L*_1_-norm of matrix *B*, representing the sum of the element’s absolute values.

Although regularizations may reduce the overfitting and generate sparse solutions, the information in the results comes only from the gene expression data. Due to the lack of data in the time dimension, the results are not stable in reality. Previous studies [31] illustrated that in the under-determined condition (e.g., *T*<(*n*+1)*p*), the linear regression solution (Lasso) is not unique, thus making it difficult to interpret the results.

To ensure that the causality network prediction is more stable and accurate, we propose to add rich biological prior knowledge to guide the solution using regularizations.

Prior knowledge in the public databases can be summarized to pairwise gene-gene relationships. In this paper, we formulate the prior knowledge about an organism as a weighted graph among the target gene set *G*=(*V*,*E*). A node set, *V*, represents the target gene set, where |*V*|=*n*. An edge set, *E*, represents the association between two different genes, which could be either directed or undirected depending on the prior knowledge. Also, *E*_*ij*_ is used to represent the weight of the edge *i*⇒*j* (typically *E*_*ij*_≥0). Since we could straightforwardly convert the undirected graph into a directed graph by setting *E*_*ij*_=*E*_*ji*_, we only discuss the directed case here.

Inspired by the idea of Bayesian priors [32], we got the idea of building a weight matrix, *W*, to guide the regularization process. First, we constructed a weight *n*×*n* matrix *W* ^′^, from the prior knowledge graph *G*=(*V*,*E*). Building prior knowledge matrix *W* ^′^ is a case-by-case process for scientists: for continuous values like TF binding scores and YeastNet association network, we use a linear mapping to build *W* ^′^ from *E*; for discrete values like protein-protein interaction, we use 0−1 binary mapping to build *W* ^′^. The guideline is that higher the absolute value of *W* *ij*′ is, the more likely there is a causality/regulatory relationship between gene node *i* and *j*. When prior knowledge does not indicate any model order information, we simply repeat the prior knowledge *n*×*n* matrix *W* ^′^ *p* times to obtain the following *n* *p*×*n* matrix *W*:

$$\underset{(np\times n)}{W}=\left[ \begin{array}{c} W'\\ W'\\ \vdots \\ W' \end{array} \right]. $$

Based on the prior knowledge matrix *W*, we can subsequently modify the optimization formula to a new form:

(13) $$  \hat{B}=\underset{B}{\operatorname{argmin}}\frac{1}{2}*||Y-XB||_{F}^{2}+\frac{1}{2}*\lambda_{1}||B-\lambda_{2}W||_{F}^{2}.  $$

It is noteworthy that the regression parameters in solution *B* might be either positive or negative, corresponding to positive or negative regulation relationships. Therefore, we employ a preliminary estimation, *B* ^∗^, resulting from ordinary Ridge regression to modify the sign of *W*_*ij*_ before *W* is incorporated into the regularization. Together, we design a new Granger causality modeling method that is regularized by prior knowledge, viz., CGC using a two-step prior knowledge Ridge regularization, termed **CGC-2SPR**:



All the Ridge or Ridge-like problems could be solved by convex optimization strategies (Eq. 13). First, we rewrite the objective function (Eq. 13) as the following form:

(14) $$  \begin{aligned} f(B)=&\frac{1}{2}*tr((Y-XB)^{T}(Y-XB)) \\ &+\frac{1}{2}*\lambda_{1}tr((B-\lambda_{2}W)^{T}(B-\lambda_{2}W)) \end{aligned}  $$

where *f*(*B*) is the objective function, *Y* and *X*, respectively, are the observation and feature matrix which are the same as (9). *W* is the prior knowledge weight matrix generated by the aforementioned procedures.

Then we calculate a partial derivative of *f*(*B*) with respect to *B* matrix:

(15) $$  \frac{\partial f}{\partial B}=X^{T}(XB-Y)+\lambda_{1}(B-\lambda_{2}W).  $$

When $\frac {\partial f}{\partial B}=\textbf {0}$, *f* obtains its optimal (minima) solution:

(16) $$  \hat{B}=(X^{T}X+\lambda_{1}I)^{-1}(X^{T}Y+\lambda_{1}\lambda_{2}W).  $$

Other Ridge-like problems could be solved similarly.

For Lasso problems, they have a form that is similar to the second equation in (Eq. 12). We utilized GLMNET algorithm to solve Lasso optimization [33]. In the experiments with lots of genes, it turns out that the Lasso GC model is much slower than the Ridge GC model due to the *L*_1_ loss term. As a result, the Ridge-based *CGC-2SPR* is more preferred to efficiently analyze real data.

### Significance level estimation using Monte Carlo simulation

A common practice in biological research is that the final analysis results are judged and compared with statistical meaningful significance levels. However, when *n*>>*T*, the standard *F*-tests for any conditional Granger causality cannot be applied anymore as its assumption is violated. Specifically, to estimate the *F* distribution for CGC, *F*(*p*,*T*−*p*−*n*∗*p*), *T*−*p*−*n*∗*p* must be positive. However, it is always below zero when *n*>>*T*. When *T* is an extremely small number, it is very difficult to apply the Wald test [34] as well.

To solve the challenge, we propose to use Monte Carlo methods to approximate the distribution of *B*. Then based on its distribution, we are able to calculate and estimate the significance levels of the discovered causality edges and networks: Assuming there is a resulting causality edge from variable *i* (source) to *j* (target), our proposed Monte Carlo Significance Estimation (*MCSE*) algorithm is shown in Algorithm 2.



We can parallelize the *MCSE* computation to quickly estimate the level of significance. *MCSE* works like the *F*-test in the CGC model in the sense that it keeps all the other expression data besides *i* and *j* as a context during the calculation. Also, it is flexible in terms of a reference (null hypothesis *H*_0_) selection. In this study we chose the normal Ridge regression as a reference in order to decide the combined significance level from the gene expression data and prior knowledge.

## Results

### Simulation dataset experiments

**Fig. 3:**
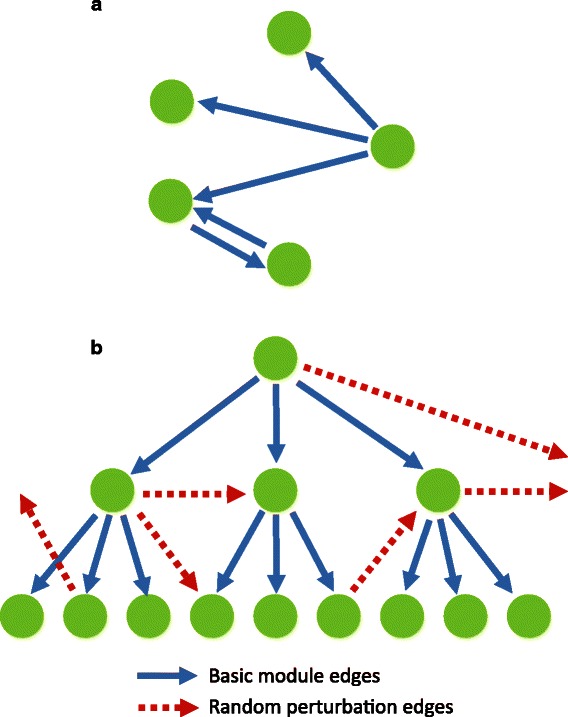
The five-variable model (**a**) was previously used in several studies [13, 24] to evaluate the performance of different gene regulatory network inference methods. However, it lacks the hierarchical modularized structure described in the real biological regulatory networks [35–37]. In this study, we generated a simulated hierarchical modularized network based on the 1 →3→9 module and the random perturbations (**b**). **a** Five-variable model. **b** Modularized hierarchical model

It is nearly impossible to obtain a real-life exemplary biology dataset with all the ground truth. In this section, we perform realistic simulation experiments to compare the performance of various causality algorithms with our proposed *CGC-2SPR*. Previous studies [13, 24] utilized the classic five-variable model (shown in Fig. 3(a)) to evaluate the performance of GRN inference methods. However, the five-variable simulation model can reflect neither the real biological regulatory networks which generally consist of hierarchical modularized sub-networks, nor real experimental data which contains many more genes than time points (*n*>>*T*). To better simulate real biological dataset, we here first generate a modularized hierarchical network, and then add simulated expression data that contain only a few time points to fit the *n*>>*T* condition. Through making these two major changes, we could better understand how the causality inference algorithms work in a more realistic dataset.

#### Modularized hierarchical simulation network generation

Real biological regulatory networks can be described to have hierarchical structures with only a few top level master regulators and many low level effectors [35–37]. Before generating the simulation networks, we first provide a few definitions: In the gene regulatory networks, gene nodes that are not regulated by others are defined as *master regulators*, while the remaining genes are called *effectors*. Notably, some *effectors* can also regulate other genes, as shown in the middle layer of Fig. 3(b).

The basis of our proposed simulation model is rooted in a simple 1 →3→9 regulatory module, representing the basic unit of the three-layer hierarchical regulatory network (Shown in Fig. 3(b)). Such a basic three-layer hierarchical regulatory module (13 genes) is repeated 60 times to create the initial network, with 13×60=780 nodes. Then, we add random perturbations edges to the initial network to create connections among different modules.

**Fig. 4:**
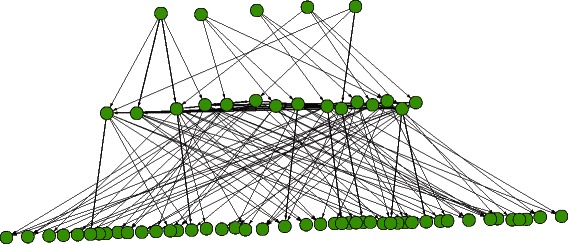
The overall hierarchical layered structure of the generated golden standard regulatory networks. For simplicity, a sample network with only 5 repeats of the basic modules is shown here

In real biological experiment, only a subset of activated genes with different expression values across time points will be selected to be analyzed. To simulate the condition, we randomly decided whether the *master regulators* are activated in the biological process and activate around half of the regulatory network. In this case, a total of 651 genes are activated and survive the filtering process. Moreover, we added 349 independent (isolated) nodes with periodic expression as a background noise to interfere with the learning. Altogether, a regulatory network with 1000 nodes and 1082 regulatory edges was generated for the simulation purpose. A simpler network with only five repeats is shown in Fig. 4, representing the modularized hierarchical layout of the actual simulation network. The final network will be used as the golden standard for both expression data generation and the evaluation criteria for different causality inference methods.

#### Gene expression data generation

Guided by the golden standard regulatory network generated from the previous step, we used a linear model to obtain the time series gene expression data. The expression values of *master regulators* and *effectors* are generated differently according to the regulatory graph.

For the *master regulator* nodes, their expression values are generated from a periodic *A* *R*(2) model [38], similar to the condition of the yeast metabolic cycle (YMC) dataset used in this study.

(17) $$  {\small{\begin{aligned} {}x(j, t)=a*d(j)* x(j, t-1) + b*d(j)^{2}* x(j, t-2) +e(j,t) \\ \end{aligned}}}  $$

where the cyclic controlling coefficients are:

(18) $$  \begin{aligned} a\ =&\ exp\left(\frac{1}{6}\pi i\right) + exp\left(-\frac{1}{6}\pi i\right)=\sqrt{3} \\ b\ =&\ exp\left(\frac{1}{6}\pi i\right) * exp\left(-\frac{1}{6}\pi i\right)=1\\ \end{aligned}  $$

*x*(*j*,*t*) denotes the expression of gene *j* at time point *t*. *d*(*j*) is the decay factor of gene *j* expression, and is randomly sampled from a uniform distribution *U*(0.95,1). *e*(*j*,*t*) is the random noise that conforms to a Gaussian distribution *N*(0,1). In this case, the *master regulator*’s expression values form a periodic pattern every 12 time points due to the coefficients *a* and *b* in the *A* *R*(2) model [38].

The expression values of the *effectors* are controlled by their parents in the regulatory graph at different model orders.

(19) $$  \begin{aligned} x(k, t) =& \sum\limits_{j=1}^{n}r(j\Rightarrow k)*x(j, t-p(j\Rightarrow k)) + e(k,t)\\ \end{aligned}  $$

If gene *j* regulates gene *k*, *r*(*j*⇒*k*)∼*U*(−1,1). Otherwise, *r*(*j*⇒*k*)=0. *p*(*j*⇒*k*) represents the model order of the regulation from gene *j* to *k*, and is generated randomly from [1…3]. *e*(*k*,*t*) is the random noise that conforms to a Gaussian distribution *N*(0,1).

**Fig. 5:**
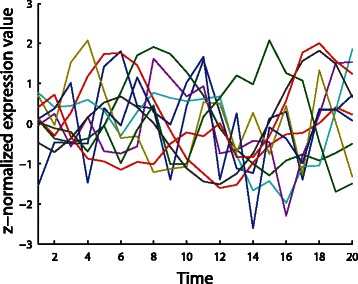
The overview of the simulation expression dataset. The simulation dataset was generated with a linear model guided by a simulated modularized hierarchical network. For simplicity, we only plotted the expression values of randomly selected 10 genes

The expression values of all the genes in the first three time points are generated randomly from a Gaussian distribution *N*(0,1). Afterward, the expression values of all the other time points are calculated by the rules described above. Altogether, only 20 time points of gene expression data have been generated from the model, similar to real biological studies. This is another major difference from previous studies which usually use hundreds of time points to train the model [13, 24]. As a last step to simulate real studies, we normalized the resulting expression data across different time points to have zero means and unit variances. The overview of the simulation expression data is shown in Fig. 5.

#### Prior knowledge graph generation

**Fig. 6:**
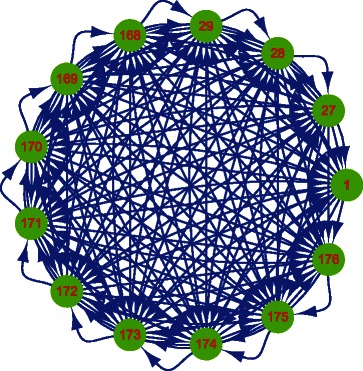
The prior knowledge structure used by *CGC-2SPR* on the simulation dataset. It consists of the 13-variable clique motif repeatedly in the basic module and could provide the group information for analyzing gene expression data

The prior knowledge graph needs to be carefully selected to confer group information to the expression data analysis. In this study, the clique graph structure in each subgroup is used as prior knowledge to represent group information. Essentially, it is a bidirectional clique graph under each 1 →3→9 regulatory unit. The basic structure of the prior knowledge graph is shown in Fig. 6. We note that the prior knowledge graph did not include random cross-module links that were added into the ground truth regulatory network. Due to the aforementioned filtering process, some of the clique regulatory relationships are not included in this prior knowledge graph.

The theoretical accuracy of the prior knowledge graph is 12/(13∗12)≈7.7 *%*. After the random activation and filtering, the actual accuracy of the remaining prior knowledge graph is about 7.5 *%*. With the help of prior knowledge, we compare the performance of *CGC-2SPR* to the standard regularization methods and other popular methods in the domain.

After establishing prior knowledge graph *E* (1000 nodes, 6748 edges), we can build the *W* matrix for *CGC-2SPR*. Firstly we convert the graph *E* to a 1000×1000 matrix form: if there is an edge from node *i* to *j*, *E*_*ij*_=1; otherwise *E*_*ij*_=0. Since the prior knowledge did not have information on the possible time lag information, we directly expanded *E* three times to obtain the *W* matrix (3000×1000). Lastly, the *W* matrix will be applied to improve Granger causality analysis by conferring group information.

#### Comparison of different methodologies

We evaluated various representative methods for analyzing time series gene expression data on the simulation dataset. In terms of regression approaches, the performance of Ridge [29], Lasso [30] and Elastic net [39] are tested on the simulation dataset. The regularization parameters were selected through cross validation. These methods were previously implemented in MATLAB using the GLMNET algorithm [33]. Moreover, pairwise GC model was also considered using the GCCA toolbox [40]. For information theory approaches, MRNET and ARACNE are used for comparisons. They are implemented in *minet* package in *R* [41]. We also tested the DBN modeling using the GeneNet ([42]). DBN generally displayed a good performance in other studies [10–12]. However, it could not complete the processing of a dataset with up to 1000 genes [13, 14] and its reported performance was similar to that of regression approaches.

To evaluate the performance of different methods, each method will generate a ranking list of the causality edges. For PGC model, the edges are ranked by their significance values. For regression methods including Ridge, Lasso, Enet and *CGC-2SPR*, the edges are ranked by the absolute values of the corresponding regression coefficients. For information theory methods, the edges are ranked by the weight calculated from the corresponding method. Afterwards, evaluations can be applied on the ranked lists generated by different models.

**Fig. 7:**
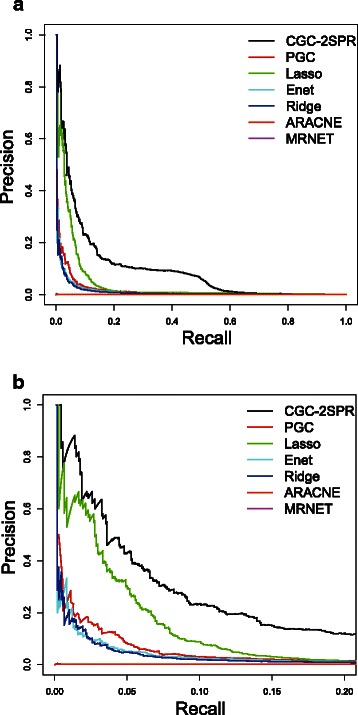
PRC plot for different methods including regression models and mutual information models. Our newly proposed *CGC-2SPR* shows significant performance improvements over all the other models. **a** PRC curve. **b** Zoomed PRC curve

Firstly, Precision-Recall (PRC) curves [43] are plotted to compare the performance of different methods. As shown in Fig. 7(a), the newly proposed method *CGC-2SPR* displays a considerable improvement over the other methods in the field. The second closest method is Lasso. However Fig. 7(a) shows that as we scan through the ranking list, Lasso generates much more false positives due to the lack of prior knowledge. Moreover, Ridge regression performed much worse than *CGC-2SPR* and even worse than PGC, thereby confirming the importance of incorporating prior knowledge. The information theoretic models could not detect regulatory relationships correctly and perform the worst among all methods.

In practice, biologists usually focus on the most significant edges. In other words, the high precision area that is on the left of the two plots in Fig. 7(a) are of more interest. Therefore, the top 1082 (edge number of the golden standard graph) causality relationships are selected by different models to calculate precision *P*, recall *R*, and then *F*_1_ score. The results are shown in Table 1.

**Table 1:** *F*_1_ score comparison for different models

Method	MRNET	ARACNE	PGC	Ridge	Lasso	Enet	CGC-2SPR
Accuracy (*F*_1_)	0.003	0.002	0.058	0.046	0.091	0.051	0.150

The results confirm again what we observed from the Precision-Recall curve: our proposed model showed 65 % performance improvement over the second best method, Lasso. Noticing that the prior knowledge itself has an accuracy of 7.5 *%*, *CGC-2SPR* actually performs better than the combination of prior knowledge and Ridge together (0.150>0.075+0.058). The additional performance is attributed to the fact that *CGC-2SPR* modeling can more effectively rule out random coincidences with the help from the group information provided by the prior knowledge.

#### Computational efficiency and scalability of CGC-2SPR

One of the major reasons that Granger causality analysis has become increasingly popular in recent years is its efficiency in comparison to other network modeling methods. Our newly developed *CGC-2SPR* is based on the Ridge regression thus is implemented with basic matrix operations (especially SVD decomposition) [44]. Asymptotically, it has the same complexity as the Ridge regression which itself is an efficient, and scalable method for inferring gene regulatory networks from thousands of genes.

For the simulation dataset used in our experiment, we tested the computation time of different methods on a server with two Intel Xeon E5-2650L CPUs and 256 GB memory. The results are listed in Table 2. Although the mutual information methods ARACNE and MRNET run faster than others, their performance is the worst in the extreme condition (*n*>>*T*). Lasso has the second best performance, but it is computationally expensive to run cross validation on Lasso, which consumes around 100 hours on a single CPU. Not only does the newly proposed *CGC-2SPR* have the best performance compared with other methods in the simulation study, it is also computationally efficient and scalable when handling thousands of genes.

**Table 2:** Real calculation time of different gene regulatory network modeling methods on the server. Since some modeling methods could be scaled up to use multi-core (multithread), we also estimated the time consumed on single thread

Method	Real time	# of threads	Estimated time
	consumed	utilized	consumed on
			single thread
MRNET	1 min	1	1 min
ARACNE	1 min	1	1 min
PGC modeling	29 mins	1	29 mins
Ridge (cross validated)	5 mins	∼16	1 hour
CGC-2SPR	5 mins	∼16	1 hour
Lasso (cross validated)	8 hours	12	96 hours
Enet (cross validated)	84 hours	12	1008 hours
DBN	NA(>1 mon)	1	NA(>1 mon)

### Biological case study on yeast metabolic network

#### Preprocessing the data

Next we applied our new method *CGC-2SPR* to a real biological dataset: “yeast metabolic cycle” (YMC) time series gene expression data. The dataset is collected from the experiments on the well-studied organism *Saccharomyces cerevisiae*, a.k.a. the baker’s yeast [28]. The corresponding GEO database [5] accession number is GSE3431. For this dataset, *n*>>*T* holds true.

**Fig. 8:**
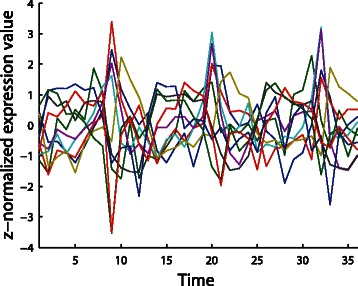
The overview of the normalized YMC expression dataset [28]. It covered three metabolic cycles of yeast in a total of 15 hours. Although only randomly selected 10 genes are plotted here, periodic patterns can be clearly observed

Published in 2005, YMC dataset consists of three metabolic cycles covering about 15 hours together. Since the gene expression measurements are taken every 25 minutes, there are 36 observations together in the time dimension (*T*=36). Through the periodicity and difference analysis, a list of periodic genes were identified with different expression levels throughout the periodic cycle [28]. Our study focuses on the periodic gene list, which consists of 2935 genes together (*n*=2935). The expression data of these periodic genes are normalized to zero-mean and unit variance. The normalized YMC expression data are shown in Fig. 8.

Also, the model order (the maximum number of lags allowed) *p* for all the approaches was chosen as two based on the results of cross validation. For a higher model order, *p*>2, the noise in the gene expression data from the selected dataset will have more impact on the results (data not shown). Nevertheless, all the approaches used in our study could deal with a higher model order when necessary.

To compare the effectiveness of different methods, we created a golden standard for evaluation from the functional transcriptional regulatory network generated in the genome wide KO (knock out) experiments [45]. Based on the genetic interactions between different genes discovered from the KO experiments, the golden standard includes direct and indirect regulatory relationships among target genes. After being filtered by the target periodic gene set dictionary, the golden standard contains 3201 causality edges which will be used to evaluate the performance of different models on the real dataset.

#### Failure of PGC model on YMC dataset

Using the golden standard as a reference, we evaluated the performance of PGC model by calculating the distribution of the significance level values (*p*-value). If the PGC model is able to recover the golden standard, the *p*-value distribution of the golden standard pairs will differ from that of all pairs in the target gene set.

We calculated the log10*p* value distribution for three types of scenarios: 1) Randomly generated time series pairs, each of which has the same time length and model order (*T*=36, *p*=2), 2) Randomly selected time series pairs from the target gene set, and 3) time series of all genes pairs from the golden standard.

**Fig. 9:**
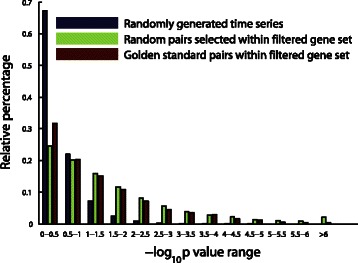
The failure of PGC model on the YMC dataset. Evaluated by the golden standard, although PGC model could distinguish golden standard from totally random generated time series, it could not distinguish the golden standard out from the random pairs in the filtered 2935-gene set

Figure 9 shows that PGC is able to distinguish the golden standard (Scenario 3) from time series data randomly generated from a Gaussian or uniform distribution (Scenario 1), but fails to differentiate the golden standard from the target gene set average (Scenario 2) by a significant margin. The main reason why PGC model fails is that when *n*>>*T*, there is a high chance that a random pair coincidentally gets low *p*-value from the PGC calculation, and so becomes a false positive. This type of failure also happens to other pairwise based models when applying to a dataset with *n*>>*T* property.

#### Building a prior knowledge graph

Different prior knowledge might have different levels of effectiveness on the inference of a causality relationship. In this paper, two types of prior knowledge for yeast were considered independently to improve the Ridge regression: the first is “YeastNet” [46], a general functional gene association network, and the second is the transcriptional factor (TF) binding profiles of yeast genes [47]. The second type of prior knowledge is more specific for improving the inferences of gene regulatory networks than the first one.

The first prior knowledge is “YeastNet”, generated by summarizing heterogeneous knowledge from various traditional biological experiments [46]. In this study, we directly used its functional association score to generate a prior-knowledge weight matrix *W*. Since “YeastNet” is an undirected graph, in the prior knowledge matrix *W*[*i*,*j*]=*W*[*j*,*i*]. After the prior knowledge graph is filtered through the dictionary of target gene set, it contains 33583 number of gene pairs, and then is used to generate a prior knowledge weight matrix *W* with 33,583×2(undirected) ×2(model order) =134332 non-zero entries.

The other prior knowledge is the genome wide TF binding profiles of the yeast genes [47], which is based on the measurements of the binding specificity from 89 yeast transcription factors to “*k*-mer” motifs. The table S5 in the study [47] is the “total occupancy scores” calculated by “median intensity *k*-mer sums”, which indicate the preferences of 85 TFs binding to different genes. We directly used these scores to build the prior knowledge matrix *W*. After target gene set dictionary based extraction, the prior knowledge graph contains information about 45(the number of TFs) ×2921(the number of the target genes) =131,445 directed gene pairs, which correspond to a prior knowledge weight matrix *W* with 131,445×1(directed) ×2(model order) =262890 non-zero entries.

#### CGC-2SPR on YMC dataset

As mentioned in the methodology section, we applied *CGC-2SPR* to the dataset in a two-step process.

Firstly, a normal Ridge regression is optimized and applied to the dataset. The optimal *λ* value in Eq. 12 obtained from cross validation is 0.01. The MSE of Ridge regression at *λ*=0.01 is much better than that of the random model that is based only on the zero regression coefficient matrix (Results not shown).

Based on the ordinary Ridge regression results (*B* ^∗^ and *λ*_1_), we started to incorporate prior knowledge into the regularization process. *λ*_1_ was chosen as 0.01, the same as the *λ* obtained from Ridge cross validation. To better mix heterogeneous knowledge, we choose the value of *λ*_2_ such that *B* is comparable to *λ*_2_ *W*: *λ*_2_=*m* *a* *x*(*B* ^∗^)/*m* *a* *x*(*W*). With this approach to set parameters, *λ*_2_ is 5*e*−3 for *CGC-2SPR* with “YeastNet” and 2*e*−6 for *CGC-2SPR* with “TF binding score”.

**Fig. 10:**
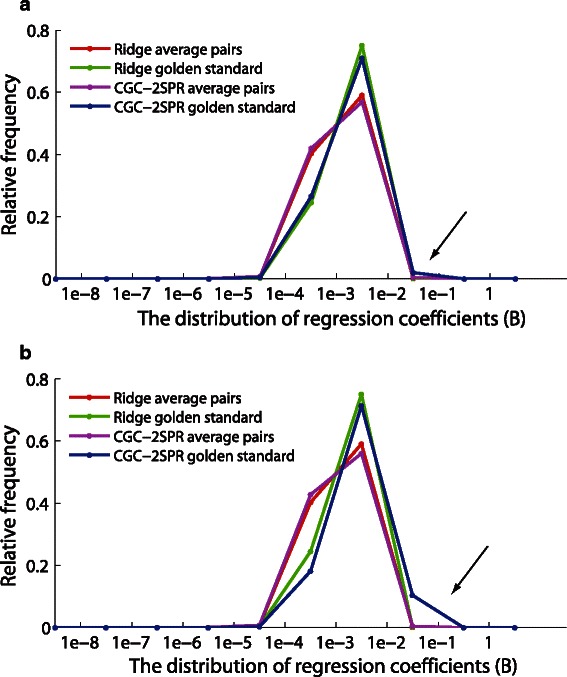
Prior knowledge “TF binding score” and “YeastNet” were separately tested on the YMC dataset using *CGC-2SPR*. The result regression coefficients (*B*_*ij*_) are sampled from either the filtered gene set (average pairs) or the golden standard. Then the normalized histograms are plotted in this figure to compare the performance of *CGC-2SPR* with different prior knowledge. “TF binding score” prior knowledge affects golden standard distribution significantly with minor effects on average pair distribution. Respectively “YeastNet” only has barely recognizable effect on the golden standard distribution. **a** CGC-2SPR using “YeastNet”. **b** CGC-2SPR using “TF binding score”

To compare the performance of *CGC-2SPR* using different types of prior knowledge, the resulting regression coefficients (*B*_*ij*_) were sampled from either the filtered gene set or the golden standard. Figure 10 shows the normalized histogram of the sampled coefficients. A model is performing well if it clearly distinguishes “golden standard pairs” from “average gene pairs within the filtered gene set” on the normalized coefficient histogram. *CGC-2SPR* with YeastNet only displays a marginal improvement that is barely recognizable with/without the prior knowledge while the average pairs are not noticeably affected (as shown in Fig. 10(a)). On the other hand, *CGC-2SPR* with the TF binding score incurs a greater boost on the gene pairs from the golden standard than those average ones in the target gene set (as shown in Fig. 10(b)). The reason that “TF binding score” provides a notable boost may be that it is directly relevant to the discovery of gene regulatory networks while “YeastNet” is mainly about the functional association between different pairs.

To quantitatively evaluate the performance of different models, we picked up the top 10000 causality entries from each model (Corresponding to the rightmost part in Fig. 10). Then we checked the overlap between the golden standards and these selected entries for different models. As Table 3 shows, *CGC-2SPR* provides significant performance improvements over the other methods. *CGC-2SPR* based on the transcriptional binding profile performs more than 20 times better than Lasso. Considering that the golden standard for real data is generally incomplete and indirect, the performance number might be even better than the number listed in Table 3.

**Table 3:** The number of golden standards in top 10000 entries

Method	PGC	MRNET	ARACNE	Ridge	Lasso	YN	CGC-2SPR(YN)	TFBS	CGC-2SPR(TFBS)
# of truth	1	5	1	7	5	20	28	87	109

YN (YeastNet) and TFBS (Transcription Factor) represent “YeastNet” and “Transcription Factor binding score” prior knowledge respectively

Moreover, the results of *CGC-2SPR* have shown that the combination of heterogeneous knowledge might achieve a “ 1+1>2” effect. For example, the RDS1 ⇒GAD1 regulatory relationship is neither discovered in Ridge, PGC, Lasso, nor on the top lists of TF binding profile. However, the newly proposed *CGC-2SPR* along with the TF binding score found the edge matching RDS1 ⇒GAD1.

#### Example networks and significance value calculations

After extracting the results from *CGC-2SPR*(TF), we analyzed the biological meanings of the discovered causality networks. Furthermore, we employed the *MCSE* algorithm to estimate the significance level of the identified causality edges.

**Fig. 11:**
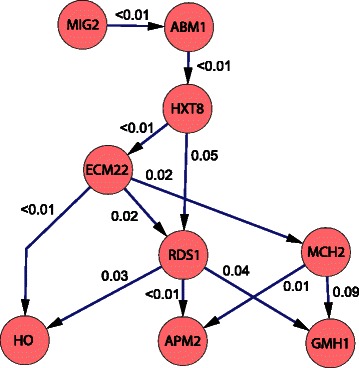
One of the discovered causality networks using *CGC-2SPR*. The edge significance values were estimated by our *MCSE* algorithm

We plotted one causality network result using Cytoscape [48], as shown in Fig. 11. The known functional annotations of these genes are taken from the *Saccharomyces* genome database [49]. MIG2 is a known Zinc finger transcription repressor, working in the glucose-induced repression of many genes. ABM1 is a protein with unknown function, but is required for normal microtubule organization. HXT8 is also a protein with unknown function, and its expression is affected by the level of glucose. ECM22 is the sterol regulatory element binding protein which regulates the transcription of sterol biosynthetic genes. When glucose is at a high level, ECM22 activates the sterol biosynthetic process that consumes glucose. HO, RDS1 and MCH2 are the downstream effector proteins that control different aspects of cell activities. In other words, the causality network shown here is involved in responding to different levels of glucose in yeast. Based on this information, we could infer that *Abm1* might be a gene that responds to different glucose levels.

## Discussion

As we observed from the simulation experiments, normal Lasso performs better than normal Ridge due to its feature selection effect. It is an interesting question whether Lasso or Elastic net with prior knowledge can provide even better performance than *CGC-2SPR*. However, the convergence properties for solving Lasso and Elastic net problems [33] are not as good as that of Ridge regression due to *L*_1_ norm, and, as a result Lasso or Elastic net is much slower than Ridge. Nevertheless, the two-step algorithm proposed in this paper is directly applicable to both Lasso and Elastic net.

How to intelligently select the right model order *p* is another interesting topic. Previous studies mentioned that choosing different model order, *p*, can significantly impact on the results [24, 25]. A higher model order generally means that more information is considered than a lower one, but potentially makes the model susceptible to noise. In reality, a moderate model order, *p*, should be chosen based on such a trade-off. We used a cross validation approach to select the right model order *p*. However, the cross validation process is computationally expensive, which prompts us to explore other efficient ways to select the appropriate model order *p* in future.

In addition, our real data experiments tested two different types of prior knowledge. This special case study indicated that closely relevant prior knowledge could assist to generate better results than general prior knowledge. Therefore, in real biological research, closely related prior knowledge is preferred whenever available.

To successfully utilize network inference methods from time series data, the corresponding biological experiments should be carefully designed. A basic requirement is that the time series should cover the whole biological phenomenon interested. For cycled biological processes, more samples in each cycle enable the possibility to discover gene regulatory relationships that happens in a smaller time scale. Also, it is preferable to have time series expression measurements that cover 1.5-2 cycles according to Nyquist-Shannon sampling theorem [50].

Last, a golden standard is usually not complete or even far from complete in real biological studies. The performance number measured by the golden standard might be lower, or even far lower than its actual performance for the real biological problem. Furthermore, the golden standard itself could also be used as a solid prior knowledge to assist the analysis of time series gene expression data. On the other hand, other types of data (e.g. genome wide TF binding data) might be noisy, but nevertheless more adequate than the golden standard, and still can better complement the expression data in discovering gene regulations.

## Conclusions

In this paper, we proposed a novel method, termed *CGC-2SPR*, that can effectively incorporate prior knowledge into Granger causality analysis, and accurately derive causal relations between gene pairs from gene expression time series data. In contrast to previous studies, we generated simulation datasets with a close-to-real condition (*n*>>*T*) and used it to evaluate previous methods and our new approach. In our simulation experiments, *CGC-2SPR* showed significantly better prediction accuracy than the other popular methods, including PGC, Ridge and Lasso regularizations, information theory approaches. For real data experiments, we applied the new method to infer causal networks from the yeast metabolic cycle dataset, along with two types of prior knowledge (TF binding profile and YeastNet) respectively. Through the golden standard data evaluation, *CGC-2SPR* demonstrated both improved performance and the advantage of combining heterogeneous knowledge. Furthermore, we proposed a new Monte Carlo method *MCSE* to estimate the significance levels of causal relations.

## Appendix

### The noise effect on the performance of network inference methods

In this section, we studied the noise effect on the performance of network inference methods that where evaluated in the manuscript, including CGC-2SPR, PGC, Ridge, Lasso, Enet, ARACNE and MRNET. The simulation datasets are generated with the same golden standard network, but at different noise levels with the Gaussian distributions *N*(0,0.25), *N*(0,0.5), *N*(0,1), *N*(0,2) and *N*(0,4) respectively.

**Fig. 12:**
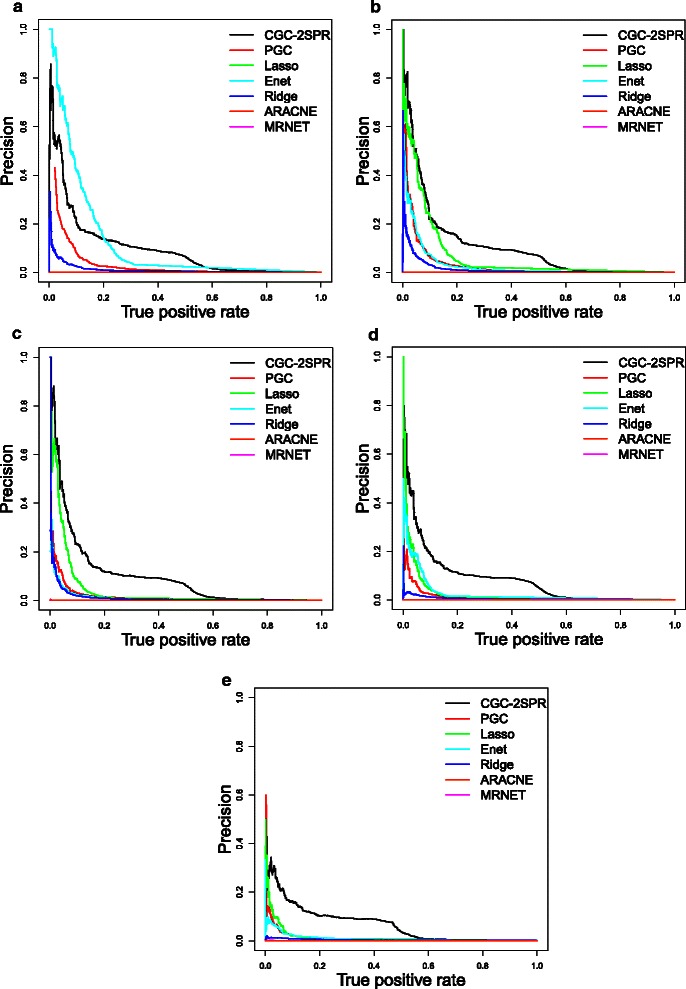
PRC plots for simulation datasets at different noise levels. In almost all the scenarios, *CGC-2SPR* has shown consistently better performance over other methods at different noise levels. Only at noise level *N*(0,0.25), Lasso and Enet (overlapping with each other in this special case) are slightly better than *CGC-2SPR*. **a** Noise N(0,0.25). **b** Noise N(0,0.5). **c** Noise N(0,1). **d** Noise N(0,2). **e** Noise N(0,4)

We evaluated the performance of the network inference methods over the generated simulated expression profiles. Fig. 12 shows the Precision-Recall (PRC) curves [43] of the results by these methods. When the simulation dataset contains a very low level of noise, i.e., *N*(0,0.25), Lasso and Enet (The best Enet obtained from cross validation is Lasso in this special case) performed better than CGC-2SPR. In all other noise levels, *CGC-2SPR* performs stably and consistently better than all the remaining methods. With the help of prior knowledge, the PRC curve of *CGC-2SPR* only slightly drops as the noise level increases.

### The performance of the time-lagged version of ARACNE and MRNET

Time-delayed version of ARACNE and MRNET has been verified in existing studies [17, 19] to be able to capture the regulatory relationship and to provide better performance than does the standard version of ARACNE and MRNET. However, these studies have involved datasets with small gene numbers (e.g. 5 nodes subnetwork) and tens of time points (32 and 45 time points after preprocessing) [19]. In this additional section, we tested the time-delayed version of ARACNE and MRNET on our simulation dataset with the noise level *N*(0,1).

The time-delayed version of MRNET (TD-MRNET) is implemented by following the descriptions in [19]: Firstly we build a mutual information matrix based on the maximum value of different time-delayed mutual information calculation and record time lag associated with the maximum mutual information; then we apply normal MRNET algorithm to the mutual information matrix to infer regulatory networks; Lastly we adjust the direction of the discovered regulatory relationships according to the time lags recorded in the first step. The TD-ARACNE proposed in [17] cannot be efficiently applied to large networks with 1000 nodes. Instead we developed a modified TD-ARACNE version similar to TD-MRNET: building the mutual information matrix with the time-delayed mutual information then apply the normal ARACNE algorithm to the matrix to generate a directed regulatory network.

**Fig. 13:**
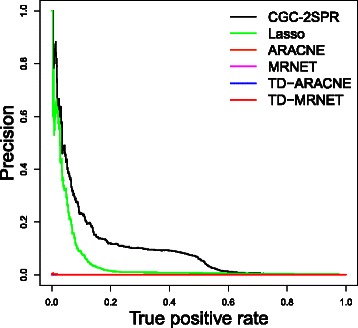
The performance of TD-ARACNE and TD-MRNET are compared to other methods described in the paper. In *n*>>*T* dataset, TD-ARACNE and TD-MRNET have shown bad performances similar to ARACNE and MRNET since they rely on pairwise mutual information calculation

We compared the performance of TD-ARACNE, TD-MRNET, ARACNE, MRNET and the two best performing methods, i.e., *CGC-2SPR* and Lasso in the paper. As shown in Fig. 13, compared to Lasso and CGC-2SPR, these mutual information based methods, including ARACNE, MRNET, TD-ARACNE and TD-MRNET, barely have the performance over zero. The major reason is that when *n*>>*T*, these pairwise information-metric based methods are quite susceptible to noise. Therefore, random coincidence regulatory relationships will dominate the results of ARACNE, MRNET, TD-ARACNE and TD-MRNET. The tests on the other noise levels have demonstrated similar results and are not shown here.

## References

[1] Mardis ER (2011). A decade’s perspective on DNA sequencing technology. Nature.

[2] Pop M, Salzberg SL (2008). Bioinformatics challenges of new sequencing technology. Trends Genet.

[3] Hecker M, Lambeck S, Toepfer S, van Someren E, Guthke R (2009). Gene regulatory network inference: Data integration in dynamic models—a review. Biosystems.

[4] Feingold E, Good P, Guyer M, Kamholz S, Liefer L, Wetterstrand K (2004). The ENCODE (ENCyclopedia of DNA elements) project. Science.

[5] Barrett T, Wilhite SE, Ledoux P, Evangelista C, Kim IF, Tomashevsky Ms (2013). NCBI GEO: archive for functional genomics data sets—update. Nucleic Acids Res.

[6] Liang S, Fuhrman S, Somogyi R, et al. Reveal, a general reverse engineering algorithm for inference of genetic network architectures. In: Pacific Symposium on Biocomputing, vol. 3. Maui, Hawaii, USA:1998. p. 18–29. 9697168

[7] Zhao W, Serpedin E, Dougherty ER (2006). Inferring gene regulatory networks from time series data using the minimum description length principle. Bioinformatics.

[8] Haider S, Pal R (2011). Inference of a genetic regulatory network model from limited time series data. Genomic Signal Processing and Statistics (GENSIPS), 2011 IEEE International Workshop On.

[9] Haider S, Pal R (2012). Boolean network inference from time series data incorporating prior biological knowledge. BMC Genomics.

[10] Kim S, Imoto S, Miyano S (2004). Dynamic bayesian network and nonparametric regression for nonlinear modeling of gene networks from time series gene expression data. Biosystems.

[11] Zou M, Conzen SD (2005). A new dynamic bayesian network (dbn) approach for identifying gene regulatory networks from time course microarray data. Bioinformatics.

[12] Zhu J, Chen Y, Leonardson AS, Wang K, Lamb JR, Emilsson V (2010). Characterizing dynamic changes in the human blood transcriptional network. PLoS Comput. Biol.

[13] Zou C, Feng J (2009). Granger causality vs. dynamic bayesian network inference: a comparative study. BMC Bioinformatics.

[14] Young WC, Raftery AE, Yeung KY (2014). Fast bayesian inference for gene regulatory networks using scanbma. BMC Syst Biol.

[15] Basso K, Margolin AA, Stolovitzky G, Klein U, Dalla-Favera R, Califano A (2005). Reverse engineering of regulatory networks in human b cells. Nat Genet.

[16] Meyer PE, Kontos K, Lafitte F, Bontempi G (2007). Information-theoretic inference of large transcriptional regulatory networks. EURASIP J Bioinformatics Syst Biol.

[17] Zoppoli P, Morganella S, Ceccarelli M (2010). Timedelay-aracne: Reverse engineering of gene networks from time-course data by an information theoretic approach. BMC Bioinformatics.

[18] Lopes FM, de Oliveira EA, Cesar RM (2011). Inference of gene regulatory networks from time series by tsallis entropy. BMC Syst Biol.

[19] Lopes M, Bontempi G (2013). Experimental assessment of static and dynamic algorithms for gene regulation inference from time series expression data. Front Genet.

[20] Granger CW (1969). Investigating causal relations by econometric models and cross-spectral methods. Econometrica: J Econometric Soc.

[21] Granger CW (1980). Testing for causality: a personal viewpoint. J Econ Dyn Control.

[22] Mukhopadhyay ND, Chatterjee S (2007). Causality and pathway search in microarray time series experiment. Bioinformatics.

[23] Nagarajan R, Upreti M (2010). Granger causality analysis of human cell-cycle gene expression profiles. Stat Appl Genet Mol Biol.

[24] Tam GHF, Chang C, Hung YS. Application of Granger causality to gene regulatory network discovery. In: Systems Biology (ISB), 2012 IEEE 6th International Conference On. Xi’an, China: 2012. p. 232–9.

[25] Lozano AC, Abe N, Liu Y, Rosset S (2009). Grouped graphical Granger modeling for gene expression regulatory networks discovery. Bioinformatics.

[26] Geier F, Timmer J, Fleck C (2007). Reconstructing gene-regulatory networks from time series, knock-out data, and prior knowledge. BMC Syst Biol.

[27] Whitfield ML, Sherlock G, Saldanha AJ, Murray JI, Ball CA, Alexander KE (2002). Identification of genes periodically expressed in the human cell cycle and their expression in tumors. Mol Biol Cell.

[28] Tu BP, Kudlicki A, Rowicka M, McKnight SL (2005). Logic of the yeast metabolic cycle: temporal compartmentalization of cellular processes. Science.

[29] Hoerl AE, Kennard RW (1970). Ridge regression: Biased estimation for nonorthogonal problems. Technometrics.

[30] Tibshirani R (1996). Regression shrinkage and selection via the lasso. J R Stat Soc Series B (Methodological).

[31] Tibshirani RJ (2013). The lasso problem and uniqueness. Electron J Stat.

[32] Zhang Y (2004). Using bayesian priors to combine classifiers for adaptive filtering. Proceedings of the 27th Annual International ACM SIGIR Conference on Research and Development in Information Retrieval.

[33] Friedman J, Hastie T, Tibshirani R (2010). Regularization paths for generalized linear models via coordinate descent. J Stat Softw.

[34] Harrell FE (2001). Regression modeling strategies: with applications to linear models, logistic regression, and survival analysis.

[35] Yan KK, Fang G, Bhardwaj N, Alexander RP, Gerstein M (2010). Comparing genomes to computer operating systems in terms of the topology and evolution of their regulatory control networks. Proc Nat Acad Sci.

[36] Cheng C, Yan KK, Hwang W, Qian J, Bhardwaj N, Rozowsky J (2011). Construction and analysis of an integrated regulatory network derived from high-throughput sequencing data. PLoS Comput Biol.

[37] Gerstein MB, Kundaje A, Hariharan M, Landt SG, Yan KK, Cheng C (2012). Architecture of the human regulatory network derived from encode data. Nature.

[38] Brockwell PJ, Davis RA (2009). Time series: theory and methods.

[39] Zou H, Hastie T (2005). Regularization and variable selection via the elastic net. J R Stat Soc Series B (Statistical Methodology).

[40] Seth AK (2010). A MATLAB toolbox for Granger causal connectivity analysis. J Neurosci Methods.

[41] Meyer PE, Lafitte F, Bontempi G (2008). minet: Ar/bioconductor package for inferring large transcriptional networks using mutual information. BMC Bioinformatics.

[42] Yeung MS, Tegnér J, Collins JJ (2002). Reverse engineering gene networks using singular value decomposition and robust regression. Proc Nat Acad Sci.

[43] Powers D (2011). Evaluation: From precision, recall and f-measure to roc., informedness, markedness & correlation. J Mach Learn Technol.

[44] Golub GH, Reinsch C (1970). Singular value decomposition and least squares solutions. Numerische Mathematik.

[45] Hu Z, Killion PJ, Iyer VR (2007). Genetic reconstruction of a functional transcriptional regulatory network. Nat Genet.

[46] Lee I, Li Z, Marcotte EM (2007). An improved, bias-reduced probabilistic functional gene network of baker’s yeast, *Saccharomyces cerevisiae*. PloS One.

[47] Zhu C, Byers KJ, McCord RP, Shi Z, Berger MF, Newburger DE (2009). High-resolution dna-binding specificity analysis of yeast transcription factors. Genome Res.

[48] Smoot ME, Ono K, Ruscheinski J, Wang PL, Ideker T (2011). Cytoscape 2.8: new features for data integration and network visualization. Bioinformatics.

[49] Cherry JM, Adler C, Ball C, Chervitz SA, Dwight SS, Hester ET (1998). SGD: *Saccharomyces* genome database. Nucleic Acids Res.

[50] Jerri AJ (1977). The shannon sampling theorem–its various extensions and applications: A tutorial review. Proc IEEE.

[51] Pramila T, Wu W, Miles S, Noble WS, Breeden LL (2006). The Forkhead transcription factor Hcm1 regulates chromosome segregation genes and fills the S-phase gap in the transcriptional circuitry of the cell cycle. Genes Dev.

